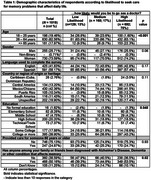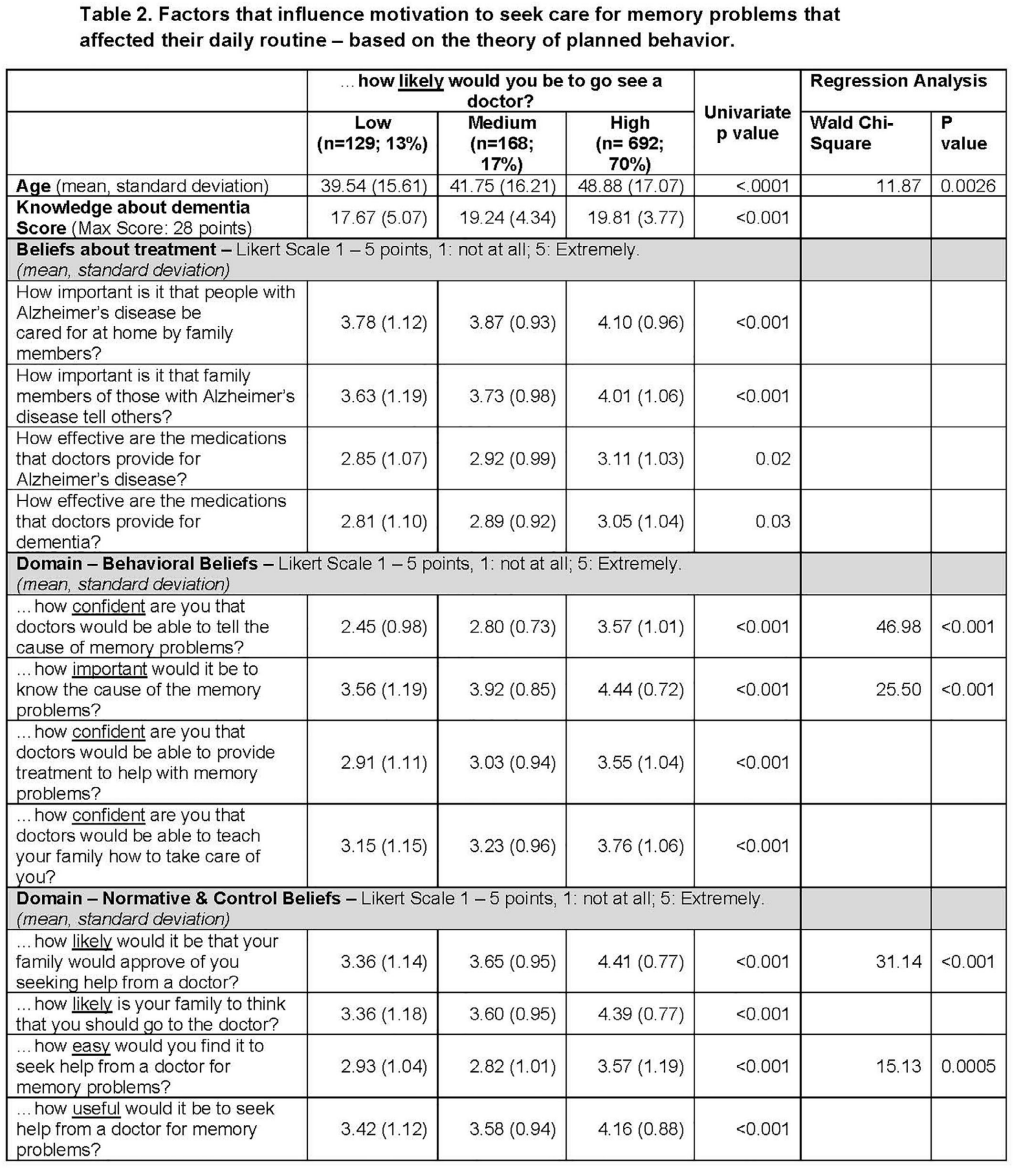# What drives Latino/a/e/x/ individuals to seek care for memory complaints: A Cross‐Sectional Study

**DOI:** 10.1002/alz70860_106924

**Published:** 2025-12-23

**Authors:** Maria C Mora Pinzon, Yelba Castellon‐Lopez, Susie Fernandez De Cordova, Maria del Carmen Rosales, Javier Neira Salazar, George Levy, Valentina B Flores Diaz, Diana C Martinez Garcia, Philip Sayegh, Cynthia M. Carlsson

**Affiliations:** ^1^ Wisconsin Alzheimer's Institute, University of Wisconsin ‐ Madison, Madison, WI, USA; ^2^ Department of Medicine, Division of Geriatrics, School of Medicine and Public Health, University of Wisconsin‐Madison, Madison, WI, USA; ^3^ Cedars‐Sinai Medical Center, West Hollywood, CA, USA; ^4^ University of Wisconsin ‐ Madison, School of Medicine and Public Health, Madison, WI, USA; ^5^ University of Wisconsin School of Medicine and Public Health, Madison, WI, USA; ^6^ School of Medicine and Public Health, University of Wisconsin ‐ Madison, Madison, WI, USA; ^7^ University of Wisconsin ‐ Madison, Madison, WI, USA; ^8^ University of California, Los Angeles, Los Angeles, CA, USA; ^9^ Wisconsin Alzheimer's Institute, University of Wisconsin, Madison, WI, USA, Madison, WI, USA; ^10^ Department of Medicine, University of Wisconsin School of Medicine and Public Health, Madison, WI, USA; ^11^ VA Geriatric Research, Education and Clinical Center (GRECC), William S. Middleton Memorial Veterans Hospital, Madison, WI, USA

## Abstract

**Background:**

Memory loss can significantly impact an individual's quality of life, prompting many to seek professional care. Understanding the motivations behind this decision is crucial for developing effective interventions and outreach strategies. Among Latino individuals, cultural, social, and personal factors play a significant role in the decision to seek memory care. The purpose of this study was to explore these motivations to seek care in a diverse sample of Latino/a/e/x individuals living in the US, to better understand their unique challenges and develop tailored strategies to encourage timely memory care.

**Methods:**

A validated survey was administered in the United States and Puerto Rico between 2021 and 2024. The study targeted self‐identified Latino/a/e/x adults aged 18 and older. The survey consisted of 47 items, utilizing a 5‐point Likert scale to evaluate knowledge about dementia, and the domains of the theory of planned behavior (behavioral beliefs, normative beliefs, and perceived control). Data were summarized using descriptive statistics, Chi‐square tests, and the non‐parametric Mann‐Whitney U test. A multinomial logistic regression analysis was used to determine factors that were associated with the intention to seek care.

**Results:**

The survey was completed by 1,019 Latino/a/e/x individuals, 74% of whom were women, with an average age of 46.2 years (range 18–94) (Table 1). Overall, 70% of participants reported being very or extremely likely to see a doctor if they were experiencing memory problems that affected their daily routine (Table 1). The final model identified the following factors as associated with the intention to seek care: age, confidence that doctors would identify the cause, importance of knowing the cause, family approval, and ease of seeking help (Table 2). The model fit statistics indicated a good fit, with a ‐2 Log L of 1030.949 and an AIC of 1054.949.

**Conclusions:**

The results of our study show that for Latino/a/e/x individuals, the decision to seek care is directly related to age, family approval, and the desire to learn the cause of the symptoms. Future research should explore what factors affect the ease of access to services as their inclusion in standard care could incentivize timely access to healthcare services.